# An Infrastructureless Approach to Estimate Vehicular Density in Urban Environments

**DOI:** 10.3390/s130202399

**Published:** 2013-02-11

**Authors:** Julio A. Sanguesa, Manuel Fogue, Piedad Garrido, Francisco J. Martinez, Juan-Carlos Cano, Carlos T. Calafate, Pietro Manzoni

**Affiliations:** 1 DIIS, University of Zaragoza, Ciudad Escolar s/n, Teruel 44003, Spain; E-Mails: juliosanguesa@gmail.com (J.A.S.); mfogue@unizar.es (M.F.); piedad@unizar.es (P.G.); 2 DISCA, Universitat Politècnica de València, Camino de Vera s/n, Valencia 46022, Spain; E-Mails: jucano@disca.upv.es (J.C.C.); calafate@disca.upv.es (C.T.C.); pmanzoni@disca.upv.es (P.M.)

**Keywords:** vehicular networks, vehicular density estimation, warning message dissemination, VANETs

## Abstract

In Vehicular Networks, communication success usually depends on the density of vehicles, since a higher density allows having shorter and more reliable wireless links. Thus, knowing the density of vehicles in a vehicular communications environment is important, as better opportunities for wireless communication can show up. However, vehicle density is highly variable in time and space. This paper deals with the importance of predicting the density of vehicles in vehicular environments to take decisions for enhancing the dissemination of warning messages between vehicles. We propose a novel mechanism to estimate the vehicular density in urban environments. Our mechanism uses as input parameters the number of beacons received per vehicle, and the topological characteristics of the environment where the vehicles are located. Simulation results indicate that, unlike previous proposals solely based on the number of beacons received, our approach is able to accurately estimate the vehicular density, and therefore it could support more efficient dissemination protocols for vehicular environments, as well as improve previously proposed schemes.

## 1. Introduction

The convergence of wireless telecommunication, computing, and transportation technologies facilitates that our roads and highways can be both our transportation and communication platforms. These changes will completely revolutionize when and how we access services, communicate, commute, entertain, and navigate, in the coming future. Vehicular Networks (VNs) are wireless communication networks that support cooperative driving among communicating vehicles on the road. Vehicles act as communication nodes and relays, forming dynamic vehicular networks together with other nearby vehicles. VNs involve vehicle-to-vehicle (V2V) [1] and vehicle-to-infrastructure (V2I) [2] communications, and have received a remarkable attention in recent years.

The specific characteristics of vehicular networks favor the development of attractive and challenging services and applications. Though traffic safety has been the primary motive for the development of these networks [3–5], VNs also facilitate applications such as managing traffic flow, monitoring road conditions, environmental protection, and mobile infotainment applications [6–8]. Most of these applications could be more efficient if the protocols involved become aware of the density of vehicles at any given time [9], being able to adapt their behavior according to this factor. Thus, knowing the traffic density in vehicular scenarios is of great importance since it promotes the more efficient use of the wireless channel [10].

One issue to keep in mind when making any proposal related to vehicular networks is to study in detail how it behaves when modifying all the possible factors [11]. However, this can be a very time-consuming task, so it is recommended to focus only on the most important factors, overlooking the rest of the parameters. Fogue *et al.* [12] concluded that the most significant factors affecting communication in realistic urban environments are: (i) the density of vehicles, since messages are propagated much more easily in high density scenarios than in scenarios with a low vehicle density (where messages cannot exploit the inherent multihop capabilities of VNs), and (ii) the urban topology, since the presence of buildings greatly affects the wireless signal propagation.

Traditionally, in Transportation Systems, vehicle density has been one of the main metrics used for assessing the road traffic conditions. A high vehicle density usually indicates that the traffic is congested. Currently, most of the vehicle density estimation techniques are designed for using infrastructure-based traffic information systems. Hence, these approaches require the deployment of vehicle detecting devices such as inductive loop detectors, or traffic surveillance cameras [13,14]. Consequently, these approaches do not exploit the capabilities offered by the emerging self-organizing vehicular traffic information systems, where vehicles are able to collect and process the traffic information without relying on any fixed infrastructure.

In this paper we focus on the vehicle density awareness in urban environments, and we present a solution to estimate the density of vehicles based on the number of beacons received per vehicle, and the roadmap topology. We consider that vehicles, able to precisely estimate the vehicular density in their neighborhood, can adjust their diffusion scheme according to this density. When using our density estimation proposal, an adaptive system could increase successful communication probability in sparse networks by increasing its data dissemination rate, or reduce the channel contention in high density scenarios by reducing the number of broadcast messages.

The paper is organized as follows: in Section 2 we review previous works closely related to our proposal, highlighting the similarities and differences. In Section 3 we present in detail our proposal for real-time estimation of vehicular density. In Section 4 we measure the estimated error to assess the goodness of our proposal. In Section 5 we compare our proposal with a density estimation method that only relies on the information provided by the beacons received. Finally, in Section 6 we present the main conclusions of this work.

## 2. Related Work

Despite the importance of determining the vehicular density to improve the support for vehicular network applications, so far there have not been enough studies that explored the density estimation in order to improve wireless communications in vehicular environments. Next, we will discuss the most relevant works in this field. Tyagi *et al.* [15] considered the problem of vehicular traffic density estimation, using the information provided by the cumulative acoustic signal acquired from a roadside-installed single microphone. This cumulative signal comprises several noise signals such as tire noise, engine noise, engine-idling noise, occasional honks, and air turbulence noise of multiple vehicles. Based on these distributions, they used a Bayes classifier to classify the acoustic signal segments. Using a discriminative classifier, such as a support vector machine (SVM), results in further classification accuracy gained over the Bayes classifier. This mechanism requires to deploy microphones in every street to be able to estimate the vehicular density.

Tan and Chen [13] proposed a novel approach of combining an unsupervised clustering scheme called AutoClass with Hidden Markov Models (HMMs) to determine the traffic density state in a Region Of Interest (ROI) of a road in a traffic video. This approach requires to deploy video cameras in every street to be able to estimate the vehicular density, and it involves huge computational requirements.

Shirani *et al.* [10] presented the Velocity Aware Density Estimation (VADE) approach. In VADE, a car estimates the density of neighboring vehicles by tracking its own velocity and acceleration pattern. An opportunistic forwarding procedure, based on VADE estimation, was also proposed. In this procedure, data forwarding is done when the probability of having a neighbor is high, which dramatically reduces the probability of messages being dropped. This approach can be very inaccurate since the own velocity and acceleration pattern of a vehicle traveling in a city do not seem very representative when accounting for the vehicular density in the nearby roads.

Artimy [16] proposed a scheme that allows vehicles to estimate the local density, and distinguish between the free-flow and the congested traffic phases. The density estimation is used to develop a dynamic transmission-range assignment (DTRA) algorithm that adjusts the vehicle transmission range dynamically, according to the local traffic conditions. Similarly to the previous work, the scheme presented in this paper is based on the flow-density relationship, which seems to be only applicable to simple topologies such as highways. Maslekar *et al.* [9] claimed that clustering has demonstrated to be an effective concept to implement the estimation of vehicular density in the surroundings. In this work, they proposed a direction based clustering algorithm with a clusterhead switching mechanism. This mechanism aims at overcoming the influence of overtaking within the clusters. The proposed algorithm facilitates the attaining of better stability, and thus improves the density estimation within the clusters. Simulation results showed that the proposed clustering algorithm is rendered stability through the switching mechanism, and hence provides a better accuracy in terms of density estimation. However, due to high mobility, a stable cluster within a vehicular framework is difficult to implement.

Stanica *et al.* [17] considered that the medium access control protocol of a future vehicular ad-hoc network is expected to cope with highly heterogeneous conditions. An essential parameter for protocols issued from the IEEE 802.11 family is the minimum contention window used by the back off mechanism. While its impact has been thoroughly studied in the case of wireless local area networks, the importance of the contention window has been somehow neglected in the studies focusing on vehicle-to-vehicle communication. In this paper, authors showed that the adjustment of the minimum contention window depending on the local node density can notably improve the performance of the 802.11 protocol. Moreover, they compared through simulation in a realistic framework five different methods for estimating the local density in a vehicular environment, presenting the advantages and the shortcomings of each of them. Venkata *et al.* [18] proposed a clustering approach for traffic monitoring and routing, where the Cluster Head (CH) election is done based on distance and direction information. Since clusters are formed all along the road, CH's will take the responsibility of routing the message to the destination. Simulation results showed better stability, accurate density estimation in the cluster, better end-to-end delay, and good packet delivery ratio. However, the density estimation mechanism operation is limited to the vehicles within the cluster.

Other authors use the Kalman filtering technique for the estimation of traffic density. For example, Balcilar and Sonmez [19] estimate traffic density based on images retrieved from traffic monitoring cameras operated by the Traffic Control Office of Istanbul Metropolitan Municipality. To this end, they use a Kalman filter-based background estimation, which can efficiently adapt to environmental factors such as light changes. However, this approach requires the density estimation procedures to be applied to the road areas manually marked beforehand. More recently, Anand *et al.* [20] proposed a method that also uses the Kalman filtering technique for estimating traffic density. In particular, they propose using the flow values measured from video sequences and the travel time obtained from vehicles equipped with a Global Positioning System (GPS). They also report density estimation using flow and Space Mean Speed (SMS) obtained from location-based data, using the Extended Kalman filter technique.

All of these works established the importance of vehicular density awareness for neighboring areas, but none has deepened in the analysis of the accuracy of the method used to estimate this density, the best time period to gather the required data, or the effect of the topology in the results obtained. In most cases, this estimation does not take place in real time or requires infrastructure deployment. Moreover, most of the works regarding the use of Vehicular Networks only use the number of beacons to estimate the vehicular density. In this paper, we demonstrate how existing approaches can be highly inaccurate, since the characteristics of the simulated roadmap can significantly affect the obtained results, making the estimation erroneous.

## 3. Real-Time Vehicular Density Estimation

The main objective of this paper is to propose a mechanism that allows estimating the density of vehicles in a specific area by using Vehicular Networks. In particular, we intend to estimate the vehicular density taking into account the number of beacons received and the topological features of the selected area (which can be obtained from the in-vehicle GPS unit).

Our method consists of three phases. In the first phase, we first analyze the features of different cities (see Section 3.1). During the second phase, the vehicles obtain the number of beacons received (see Section 3.2). Finally, in the third phase, each vehicle can estimate the vehicular density in its neighborhood by applying an equation that requires as input parameters the values, in terms of roadmap complexity and beacons received, obtained in the previous phases. Next subsections present the different phases of our mechanism.

### 3.1. Phase 1: Features of the Cities Studied

An important issue to our vehicular density estimation approach is to obtain the different features of each roadmap (e.g., the number of streets, the number of junctions, the average distance of segments, and the number of lanes per street).

**Figure 1.**
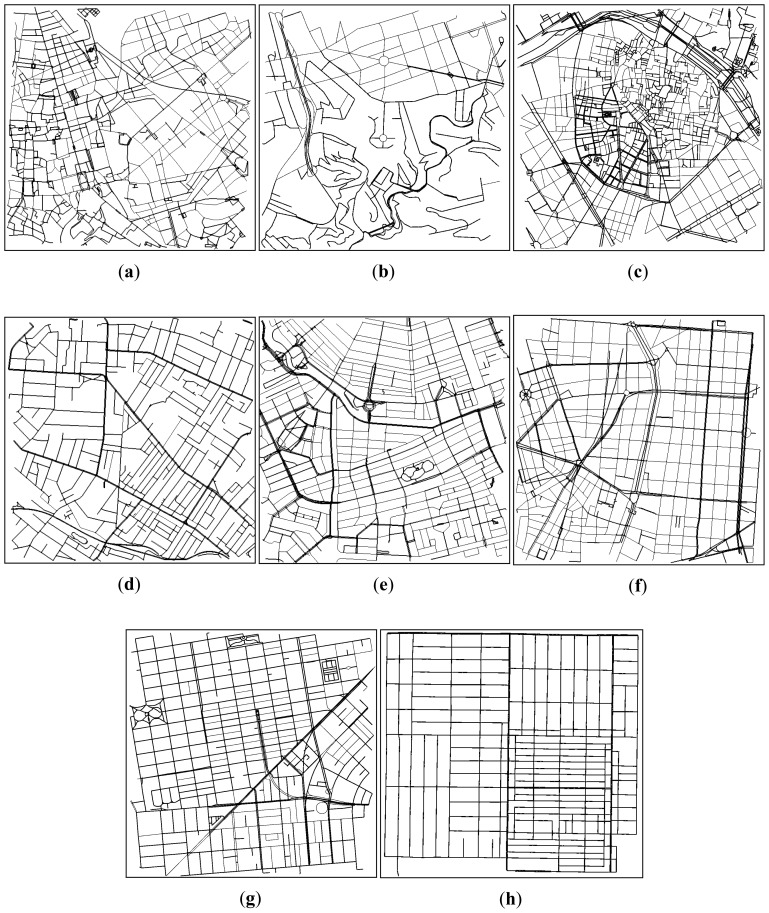
Scenarios used in our simulations. Fragments of the cities of: (**a**) Rome (Italy), (**b**) Rio de Janeiro (Brazil), (**c**) Valencia (Spain), (**d**) Sydney (Australia), (**e**) Amsterdam (Netherlands), (**f**) Madrid (Spain), (**g**) San Francisco (USA), and (**h**) Los Angeles (USA).

The roadmaps used to achieve the density estimation were selected in order to have different profile scenarios (*i.e.*, with different topology characteristics). We studied eight different cities (San Francisco, Valencia, Rome, Rio de Janeiro, Sydney, Amsterdam, Madrid, and Los Angeles). Figure 1 shows the topologies of the cities studied. Although some differences can be visually perceived, a more thorough analysis must be performed to determine and classify the topology of each map.

Table 1 shows the main features of each map of the cities under study (*i.e.*, the number of streets according to the RAV radio propagation model [21], where the visibility between vehicles is taken into consideration when identifying the different streets, the number of junctions, the average distance of segments, and the number of lanes per street.

We consider that the parameters that better correlate with the complexity of the roadmap are the number of streets and the number of junctions. Hence, we also added a column labeled as *SJ Ratio*, which represents the result of dividing the number of streets between the number of junctions. As shown, the first 5 cities (Rome, Rio, Valencia, Sydney, and Amsterdam) present an SJ ratio greater than 1, which indicates that they have a complex topology, while the rest of the cities (Madrid, San Francisco, and Los Angeles) present a lower SJ value, which indicates that they have a simple topology. Note that, although Rio de Janeiro has a relatively small number of streets and junctions, it has a complex topology since its SJ Ratio is greater than 1.

### 3.2. Phase 2: Counting the Number of Beacons Received

After performing the topological analysis of the studied maps, we need to obtain the number of beacons received by each vehicle during a certain period of time. This period is very important, since it will affect the number of beacons received, and the accuracy of the vehicular density estimation.

According to the results obtained in Section 3.3.1. in our scheme, we obtain the number of beacons received during 30 seconds. We consider that each vehicle sends one beacon per second, and that these messages, unlike warning messages, are not disseminated by the rest of the vehicles. These considerations can be found in many previous Vehicular Network studies, so they could be considered quite realistic.

The simulation results shown in this article have been obtained using the ns-2 simulator [22], modified to consider the IEEE 802.11p standard (All these improvements and modifications are available in http://www.grc.upv.es/software/). In terms of the physical layer, the data rate used for packet broadcasting is 6 Mbit·s^−1^, as this is the maximum rate for broadcasting in 802.11p. The MAC layer was also extended to include four different priorities for channel access. Therefore, application messages are categorized into four different Access Categories (ACs), where AC0 has the lowest and AC3 the highest priority.

The purpose of the 802.11p standard is to provide the minimum set of specifications required to ensure interoperability between wireless devices when attempting to communicate in potentially fast-changing communication environments. For our simulations, we chose the IEEE 802.11p because it is expected to be widely adopted by the industry.

We tested our model by evaluating the performance of a Warning Message Dissemination mechanism, where each vehicle periodically broadcasts information about itself, or about an abnormal situation (icy roads, traffic jam, *etc.*). In order to mitigate the broadcast storm problem [23], our simulations use the enhanced Message Dissemination based on Roadmaps (eMDR) scheme [24]. The eMDR scheme only allows forwarding messages when the distance between sender and receiver is greater than a threshold, or in situations where the receiver is the closest vehicle to a junction, and rebroadcasting could allow the message to reach vehicles in adjacent streets.

As for vehicular mobility, it has been obtained with CityMob for Roadmaps (C4R) [25], a mobility generator able to import maps directly from [26], and make them available for being used by the ns-2 simulator. Table 2 shows the parameters used for the simulations. All the results represent an average of over 50 repetitions with different scenarios (maximum error of 10% with a degree of confidence of 90%), and each simulation run lasted for 180 seconds.

**Figure 2.**
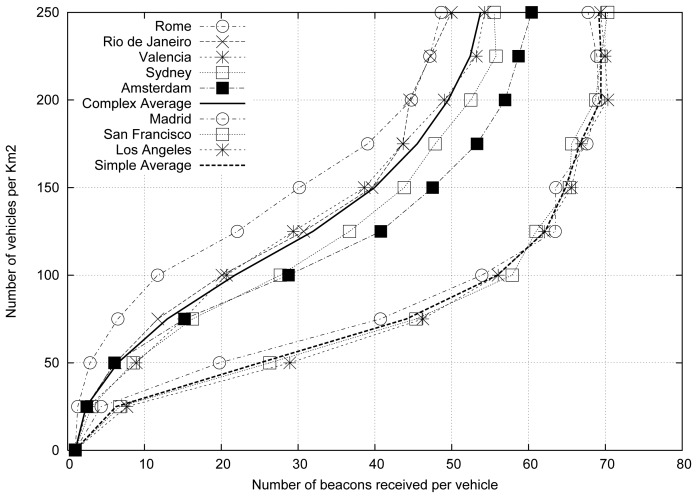
Number of beacons received when varying the vehicular density.

Figure 2 shows the results obtained for the different cities studied. We also included two lines that depict the average values for each profile category (*i.e.*, simple and complex average). As shown, two different groups can be distinguished: (i) the complex maps, which are located in the left part of the figure, and (ii) the simple maps, which are located in the right part of the figure.

As expected, complex roadmaps present a number of beacons received lower than simple roadmaps for a similar vehicular density. In addition, we found that the simpler cities present a high similitude in terms of results, being more difficult to estimate the vehicular density in complex cities compared with simple cities. Figure 2 demonstrates that the vehicular density depends not only on the number of beacons received, but also on the characteristics of the roadmap where the vehicles are located. Therefore, the characteristics of the roadmap will be very useful in order to accurately estimate the vehicular density in a given scenario. According to data shown in Table 1, the SJ ratio can be used to characterize the different maps.

Table 3 shows the average percentage difference with respect to the mean value. From the obtained results we observe that the cities that show a better fit for the average results are Valencia (in the complex topology group) and San Francisco (in the simple topology group). Hence, these cities could be used as reference to obtain representative results when simulating Vehicular Networks.

### 3.3. Phase 3: Density Estimation Function

After observing the direct relationship between the topology of the maps, the number of beacons received, and the density of vehicles, we proceed to obtain a function to estimate, with the minimum possible error, each of the curves shown in Figure 2.

To propose a method able to accurately estimate the density of vehicles, based on the number of beacons received and the roadmap topology, we made a total of 4,000 experiments. These experiments involved the simulation of controlled scenarios (*i.e.*, scenarios where the actual density is known). According to the results obtained, we propose a density estimation function capable of estimating the vehicular density in every urban environment, at any instant of time.

In order to obtain the best approach, we have tested some different functions (exponential, logarithmic, *etc.*). To this purpose, we performed a regression analysis [28] that allowed us to find the polynomial equation offering the best fit to the data obtained through simulation. Equation (1) shows the density estimation function, which is able to estimate the number of vehicles per km^2^ in urban scenarios, according to the number of beacons received, and the SJ ratio (*i.e.*, streets/junctions).

(1) $$f\left(x,y\right)=a+\text{bx}+\text{cy}+{\text{dx}}^{2}+{\text{fy}}^{2}+{\text{gx}}^{3}+{\text{hy}}^{3}+\text{ixy}+{\text{jx}}^{2}y+{\text{kxy}}^{2}$$

**Figure 3.**
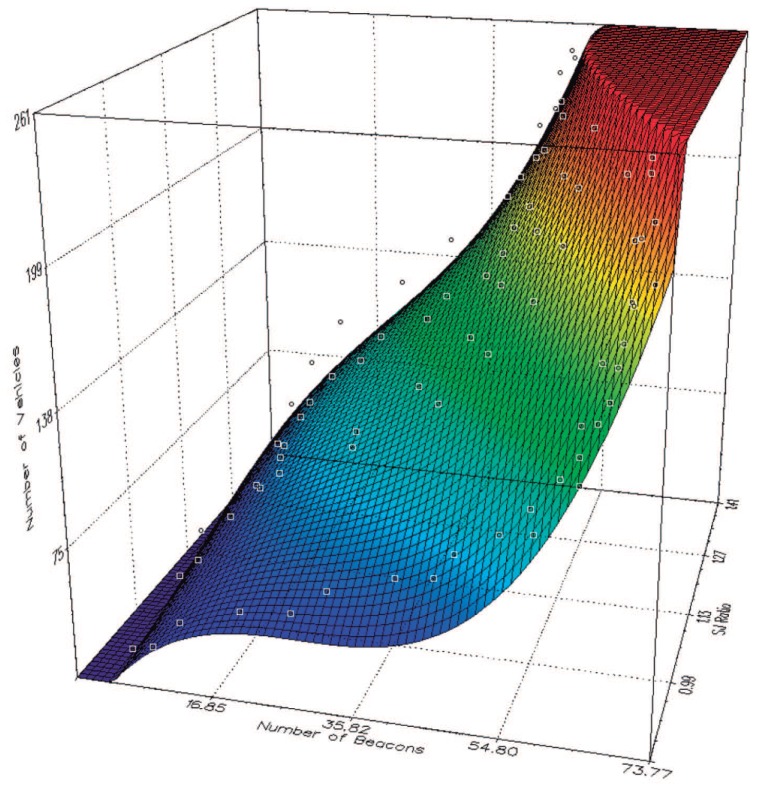
3D representation of our density estimation function.

In this equation, *x* is the number of beacons received by each vehicle, and *y* is the SJ ratio obtained from the roadmap. The values of the polynomial coefficients (*a*, *b*, *c*, *d*, *f*, *g*, *h*, *i*, *j*, *and k*) are listed in Table 4, and Figure 3 shows the 3-dimensional representation of the proposed equation.

Equation (2) presents the best non-polynomial approach we obtained. However, in terms of accuracy, the sum of squared absolute error of this function is of 3.8618E+04, while the polynomial function presents a lower value (6.3321E+03). Thus, we considered to use the first equation in our approach.

(2) $$f\left(x,y\right)=\frac{16a\cdot \text{exp}\left(-\left(\frac{x-b}{c}\right)-\left(\frac{y-d}{f}\right)\right)}{{\left(1+\text{exp}\left(-\left(\frac{x-b}{c}\right)\right)\right)}^{2}\cdot {\left(1+\text{exp}\left(-\left(\frac{y-d}{f}\right)\right)\right)}^{2}}$$

#### 3.3.1. Time Period Analysis

As mentioned before, our proposal is based on two key factors: (i) the roadmap topology, which is provided by the in-vehicle GPS systems, and (ii) the number of beacons received at a given period of time. Hence, in a vehicular density estimation system, it is very important to decide how much time is dedicated to gather important and necessary data in order to better estimate the density of vehicles at any given time.

In order to determine the optimal period of time that should be considered to estimate the density in vehicular environments, thereby enhancing the performance of the density estimation process, we made a total of 600 experiments including six different time periods (*i.e.*, 10, 20, 30, 60, 120, 180 seconds), and using the maps of Valencia and San Francisco.

**Figure 4.**
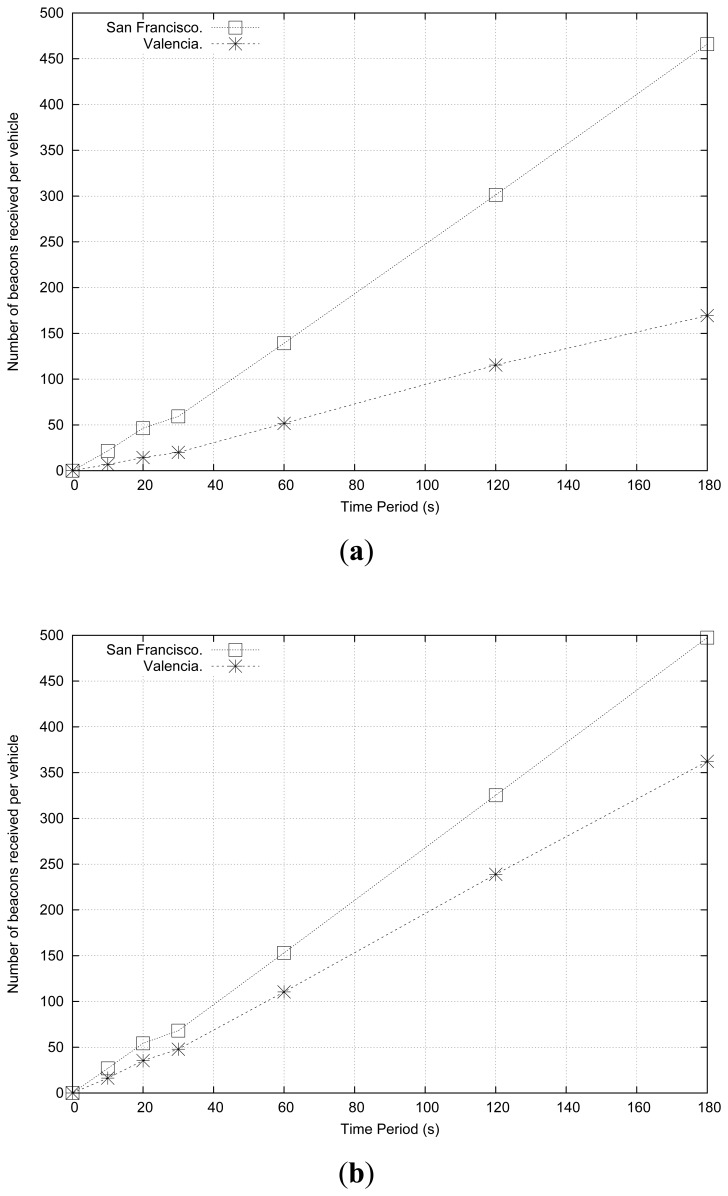
Number of beacons received per vehicle when varying the time period and the city roadmap when simulating: (**a**) 100 vehicles·km^−2^, and (**b**) 200 vehicles·km^−2^.

Figure 4 shows the number of beacons received by each vehicle when simulating 100 and 200 vehicles·km^−2^, respectively. As shown, complex maps are more affected by vehicular density variations, since messages encounter more difficulties to be propagated in these kinds of maps, especially in lower density scenarios. Regarding the optimal time period, since results are quite linear, a larger time period seems to be the best option; notice that this solution requires fewer calculations, thereby reducing the overhead. However, a more thorough analysis should be made to determine the optimal time period required to gather the number of beacons received.

To find the best period, we also analyzed the error committed when using different time periods. Specifically, we fitted the function coefficients to each period, and then calculated the absolute error committed.

Table 5 shows the median and the variance of the absolute error for each period analyzed. As shown, lower periods obtain more accurate results. In fact, when the time period exceeds 30 seconds, the error increases by two orders of magnitude. Having discarded larger periods, we consider that the best period to gather data is of 30 seconds, since the absolute error offers a lower variance.

### 3.4. The Concept of Street

Our vehicular density estimation approach uses three different parameters: (i) the number of beacons received, (ii) the number of junctions, and (iii) the number of streets. As for the number of junctions, it is only necessary to count the junctions between different street segments. However, regarding the number of streets, we realized that different alternatives could be selected to obtain the number of streets in a given roadmap.

Basically, the different alternatives are: (i) the number of streets obtained in SUMO [29], where each segment between two junctions is considered a street, (ii) the number of streets obtained in [26] (OSM), where each street has a different “name”, and (iii) the number of streets according to the RAV radio propagation model [21], where the visibility between vehicles is taken into consideration when identifying the different streets.

**Figure 5.**
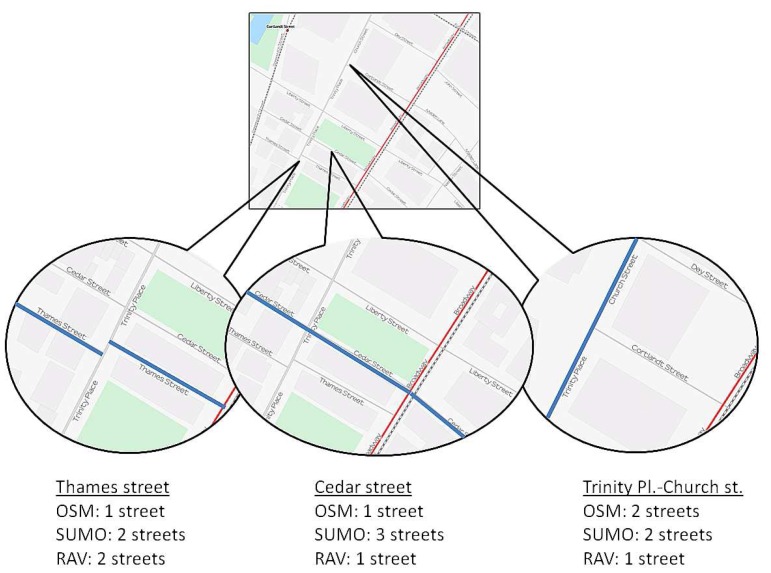
Different criteria when counting the number of streets.

Figure 5 shows a small portion of New York City to depict the different criteria when counting the number of streets. For example, Thames Street is considered only one street in OSM, whereas the SUMO and RAV models consider that there are two different streets instead. However, if we observe Cedar Street, the RAV visibility model and the OSM approaches consider a single street (as expected), whereas it is represented by three different streets according to SUMO, since it has three different segments. Finally, according to both the OSM and SUMO approaches, Trinity Place and Church Street are represented as two different streets, whereas the RAV model considers that only one street exists.

Table 6 shows the values obtained when counting the number of streets of some of the cities studied, according to each criterion (*i.e.*, SUMO, OSM, or RAV). As shown, the differences between these approaches are significant, meaning that it is important to decide which one to use in order to obtain accurate and realistic results. After some experiments, we realized that the third approach better correlates with the real features of cities, since the other two present some drawbacks: they are not accurate enough, or they present some errors (e.g., SUMO always considers segments between junctions as streets, and using street names to estimate the communication between the vehicles may result in inaccurate estimations). So, we choose the RAV model to count the number of streets in our vehicular density estimation mechanism.

## 4. Validation of Our Proposal

**Figure 6.**
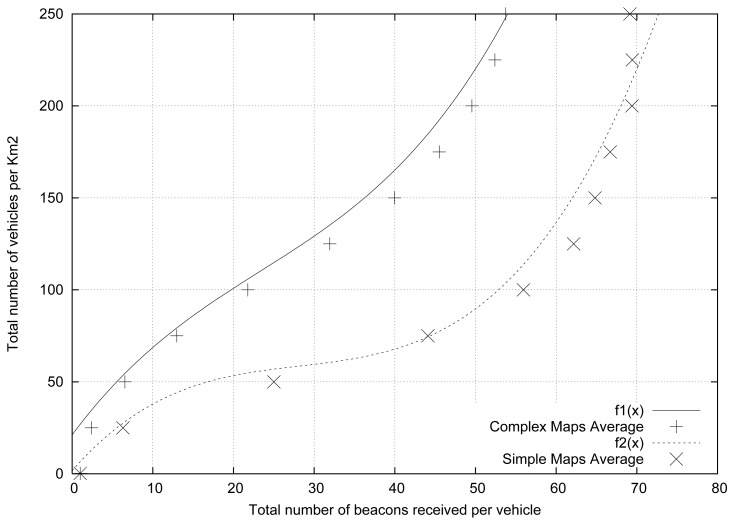
Comparison between simulated and estimated average results.

To determine the accuracy of our proposal, we proceed to measure the estimated error. Figure 6 shows the difference between the average values for all the cities studied, and the values obtained by our function. As shown, we achieve a good fit for the average values obtained in the simulations. In addition, Table 7 shows the different types of errors calculated when comparing our density estimation function with the values actually obtained. Note that the average relative error is only 1.02%.

**Figure 7.**
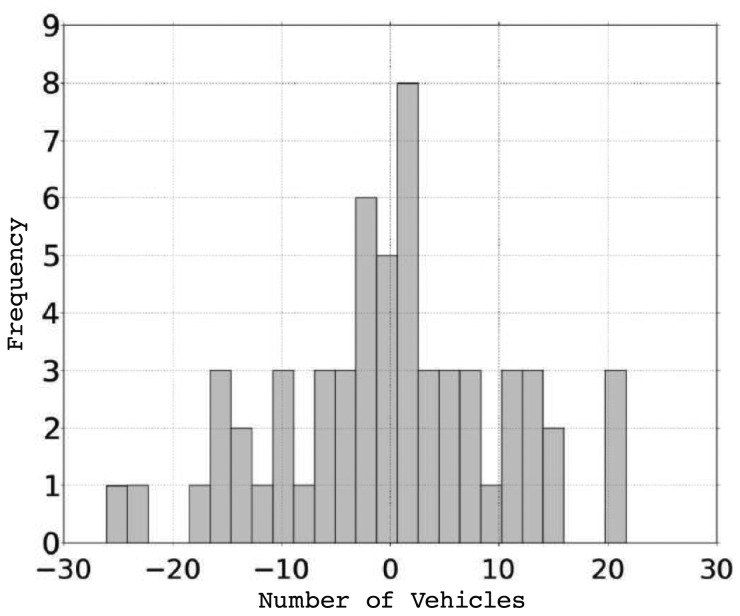
Absolute error histogram.

Finally, Figure 7 shows the absolute error histogram. As shown, results are mainly concentrated around zero, which confirms that our proposal is consistent with the expected results, and that the density estimation is accurate enough.

## 5. Comparing Our Proposal with a Beacons-Based Density Estimation Approach

As previously mentioned, other vehicular density estimation proposals rely on the use of infrastructure elements to estimate the vehicle density (e.g., [15], and [13]). On the contrary, the proposals based on V2V communications do not require the deployment of any infrastructure nodes, but they usually take into account just the number of beacons received (e.g., [9], and [17]), while omitting any data related to the map topology where the vehicles are located at.

In order to assess the importance of the topology, we compared our proposal with a beacon-based approach, where the vehicular density is estimated by only using the number of beacons received. To make a fair comparison, we followed the same methodology in both approaches.

We tested four different density estimation functions that are only based on the number of beacons received, trying to obtain the lowest sum for the squared absolute error. Specifically, we have tested three different polynomial functions (*i.e.*, quadratic, cubic, and quartic), and a non-polynomial function (based on the Preece-Baines Growth function). Equations (3)–(6) show these functions, and Table 8 shows their coefficients.

(3) $$f(x)=a+\text{bx}+{\text{cx}}^{2}$$

(4) $$f(x)=a+\text{bx}+{\text{cx}}^{2}+{\text{dx}}^{3}$$

(5) $$f(x)=a+\text{bx}+{\text{cx}}^{2}+{\text{dx}}^{3}+{\text{fx}}^{4}$$

(6) $$f(x)=\frac{a-2\cdot (a-b)}{\left(\text{exp}\left(c\cdot \left(x-d\right)\right)+\text{exp}\left(f\cdot \left(x-d\right)\right)\right)}$$

Table 9 shows the square absolute error sum for each of the functions tested. As shown, our SJ Ratio function yields more accurate results, presenting the lower sum for the square absolute error (approximately 6.332E+03, two orders of magnitude lower than the others), and it only commits an error of 8.8967 vehicles, whereas the rest of the functions that only account for the number of beacons commit an error ranging from 40.5017 to 41.5684 vehicles, depending on the selected function.

**Figure 8.**
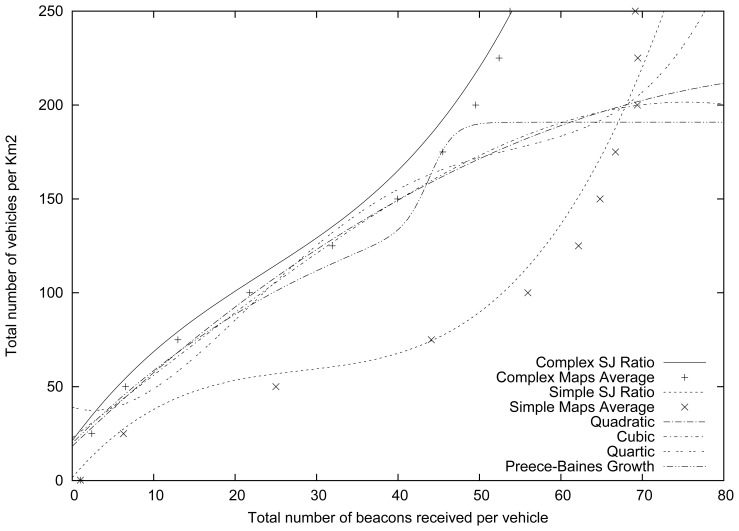
Graphical comparison between simulated and estimated results for each function.

Figure 8 shows how our approach fits well with both the Complex and Simple Maps, since it adjusts the estimation made, accounting not only for the number of beacons received, but also for the features of the maps where the vehicles are located. On the contrary, those approaches that only take into account the number of beacons received correctly estimate the density of vehicles in complex maps, specifically in low and mid-density scenarios (less than 150 vehicles·km^−2^). However, they underestimate the number of vehicles in high density environments, and, most importantly, they overestimate the density of vehicles in Simple maps.

Therefore, the advantages of using our vehicular density estimation proposal are clear in terms of accuracy. Our approach requires using GPS and digital maps, but these requirements are currently fulfilled by most of the vehicles in many countries.

## 6. Conclusions

This paper proposes a method that allows vehicles to estimate the vehicular density in their neighborhood at any given time by using Vehicular Networks. Our proposal allows scientists to improve their proposals, or propose new solutions, based on our findings.

Unlike existing proposals, our vehicular density estimation mechanism accounts not only for the number of beacons received per vehicle, but also for the map topology in the region where the vehicles are located. Our method consists of three phases: (i) we first analyze the features of different cities, (ii) the vehicles obtain the number of beacons received, and (iii) each vehicle estimates the vehicular density in its neighborhood by applying an equation that requires as input parameters the values in terms of roadmap complexity and beacons received.

Results show that our proposal allows estimating the vehicular density for any given city with a high accuracy. We also demonstrated that the characteristics of the roadmap are very useful in order to accurately estimate the vehicular density in a given scenario.

In the future, we plan to apply our proposed vehicular density estimation approach to implement more efficient and adaptive information dissemination schemes, specially designed for urban environments. We consider that, by using our density estimation proposal, an adaptive system could increase the probability of successful communication in sparse networks by increasing its data dissemination rate, or reduce the channel contention in high density scenarios by reducing the number of broadcast messages. Therefore, we plan to apply our proposal to the Profile-driven Adaptive Warning Dissemination Scheme (PAWDS) [30], which was specially designed to improve the warning message dissemination process in urban environments.

## Figures and Tables

**Table 1.** Map features.

**Map**	**Streets**	**Junctions**	**Avg. Street Length (m)**	**Lanes/Street**	**SJ Ratio**
Rome	1655	1193	77.0296	1.0590	1.3873
Rio de Janeiro	542	401	167.9126	1.1135	1.3516
Valencia	2829	2233	60.7434	1.0854	1.2669
Sydney	872	814	138.0716	1.2014	1.0713
Amsterdam	1494	1449	90,8164	1.1145	1.0311
Madrid	628	715	183.4947	1.2696	0.8783
San Francisco	725	818	171.4871	1.1749	0.8863
Los Angeles	287	306	408.2493	1.1448	0.9379

**Table 2.** Parameters used for the simulations.

**Parameter**	**Value**

roadmaps	Rome, Rio de Janeiro, Valencia, Sydney, Amsterdam, Madrid, San Francisco, and Los Angeles
number of vehicles	[100, 200, 300…1000]
number of collided vehicles	3
roadmap size	2000 *m* × 2000 *m*
warning message size	256*B*
beacon message size	512*B*
warning messages priority	*AC*3
beacon priority	*AC*1
interval between messages	1 second
MAC/PHY	802.11p
radio propagation model	*RAV* [21]
mobility model	*Krauss* [27]
channel bandwidth	6 *Mbps*
max. transmission range	400 *m*

**Table 3.** Average percentage difference with respect to the mean value.

**City**	**Percentage Difference**
Rome	29.49%
Rio De Janeiro	5.85%
**Valencia**	**4.56%**
Sydney	15.82%
Amsterdam	14.96%
Madrid	6.44%
**San Francisco**	**1.74%**
Los Angeles	4.70%

**Table 4.** Proposed equation coefficients.

**Coeff.**	**Value**
a	-1.1138191190298828E+03
b	-1.0800433554686800E+01
c	3.1832185406821718E+03
d	-4.0336415134812398E-01
f	-3.0203454502011946E+03
g	2.8542014049626700E-03
h	9.5199929660347175E+02
i	3.5319225007012626E+01
j	1.6230525995036607E-01
k	-1.6615888771467137E+01

**Table 5.** Absolute error when varying the time period.

**Time (s)**	**Median**	**Variance**
10	-5.073593E-03	1.483517E-03
20	-1.515514E-03	1.048494E-03
**30**	**-2.972316E-03**	**7.569214E-04**
60	2.377369E-01	1.241401E+03
120	7.621857E+00	1.548736E+03
180	5.128145E+00	1.492756E+03

**Table 6.** Number of streets obtained depending on the criterion used.

**City**	**SUMO**	**OSM**	**RAV**
Rome	2780	1484	1655
Rio de Janeiro	758	377	542
Amsterdam	3022	796	1494
Madrid	1387	1029	628

**Table 7.** Density estimation error.

**Error**	**Absolute**	**Relative**
Minimum	-2.612027E+01	-2.284800E-01
Maximum	2.169529E+01	5.713108E-01
Mean	-3.176197E-10	1.023340E-02
Std. Error of Mean	1.360303E+00	1.714082E-02
Median	1.698901E-01	-1.359121E-03

**Table 8.** Beacons-only functions' coefficients.

**Coefficient**	**Quadratic**	**Cubic**	**Quartic**	**Preece–Baines**
a	1.8294269144848133E+01	2.2768425534110406E+01	3.9047236513533704E+01	1.9087154795377430E+02
b	4.1367349228558270E+00	3.2941345206538704E+00	-1.3847115600040454E+00	1.6327793099067961E+02
c	-2.1509124292378768E-02	7.0289357151021746E-03	2.9758692872675790E-01	2.5673041989256740E-02
d	-	-2.5558429762904153E-04	-6.4713013709561500E-03	4.4055843266582620E+01
f	-	-	4.2741298952571685E-05	6.8666406701129157E-01

**Table 9.** Comparison between our SJ Ratio and the Beacon-based density estimation approaches.

**Fitted function**	**Sum of square absolute error**	**Vehicles error**
Beacons-only Quadratic	1.3823448453520384E+05	41.5684
Beacons-only Cubic	1.3799411801756185E+05	41.5322
Beacons-only Quartic	1.3609380737712432E+05	41.2453
Beacons-only Preece-Baines Growth	1.3123134971478261E+05	40.5017
**SJR Full Cubic**	**6.3321549647968613E+03**	**8.8967**
